# Recent Advances in Synthesis, Bioactivity, and Pharmacokinetics of Pterostilbene, an Important Analog of Resveratrol

**DOI:** 10.3390/molecules25215166

**Published:** 2020-11-06

**Authors:** Yeju Liu, Yuyang You, Juan Lu, Xi Chen, Zhihong Yang

**Affiliations:** 1 Institute of Medicinal Plant Development, Chinese Academy of Medical Sciences and Peking Union Medical College, Beijing 100193, China; yejuliu@126.com (Y.L.); jlu@implad.ac.cn (J.L.); chenxi@implad.ac.cn (X.C.); 2 School of Automation, Beijing Institute of Technology, Beijing 100081, China; youyuyang@bit.edu.cn

**Keywords:** pterostilbene, synthesis, bioactivity, pharmacokinetics, research progress

## Abstract

Pterostilbene is a natural 3,5-dimethoxy analog of resveratrol. This stilbene compound has a strong bioactivity and exists widely in *Dalbergia* and *Vaccinium* spp. Besides natural extraction, pterostilbene can be obtained by biosynthesis. Pterostilbene has become popular because of its remarkable pharmacological activities, such as anti-tumor, anti-oxidation, anti-inflammation, and neuroprotection. Pterostilbene can be rapidly absorbed and is widely distributed in tissues, but it does not seriously accumulate in the body. Pterostilbene can easily pass through the blood-brain barrier because of its low molecular weight and good liposolubility. In this review, the studies performed in the last three years on resources, synthesis, bioactivity, and pharmacokinetics of pterostilbene are summarized. This review focuses on the effects of pterostilbene on certain diseases to explore its targets, explain the possible mechanism, and look for potential therapeutic applications.

## 1. Introduction

Pterostilbene (3,5-dimethoxy-4′-hydroxy-trans-stilbene) is a trans stilbene compound with bioactivity. It was extracted and isolated from the heartwood of *Pterocarpus marsupium* for the first time in 1940 [1]. The existence of polyphenols, such as resveratrol and pterostilbene, in Darakchaava, a famous Indian herbal medicine, was studied by high performance liquid chromatography. These phenolic compounds are known antioxidants and are used in cancer chemoprophylaxis. They can reduce mortality from coronary heart disease by increasing high-density lipoprotein content and inhibiting platelet aggregation [2]. Considering these findings, resveratrol and pterostilbene have become the focus of research.

Extract from the genus *Dracaena* (Dragon’s blood) is a renowned traditional medicine in various cultures around the world [3]. Dragon’s blood extract has traditionally been used as a wound healing and antineoplastic agent because of its antibacterial, antioxidant, anti-inflammatory, and anti-apoptotic activities [4,5,6]. Resveratrol is a popular molecule that has been widely studied in recent years, and it is one of the main components of the extract of Dragon’s blood. Resveratrol and pterostilbene have similar structures, and pterostilbene is also a main bioactive compound in the extract of Dragon’s blood. Pterostilbene shows anti-inflammatory [7], anti-oxidant [8], anti-tumor [9], neuroprotective [10], lipid-lowering [11], and hypoglycemic [12] activities. Similar to other stilbene compounds, pterostilbene has two more methoxy groups on the A-benzene ring than resveratrol, indicating its higher liposolubility, which may lead to the increased permeability of the cell membrane. In many cases [11,13,14,15], pterostilbene showed significantly higher bioactivity than resveratrol.

**Figure 1:**
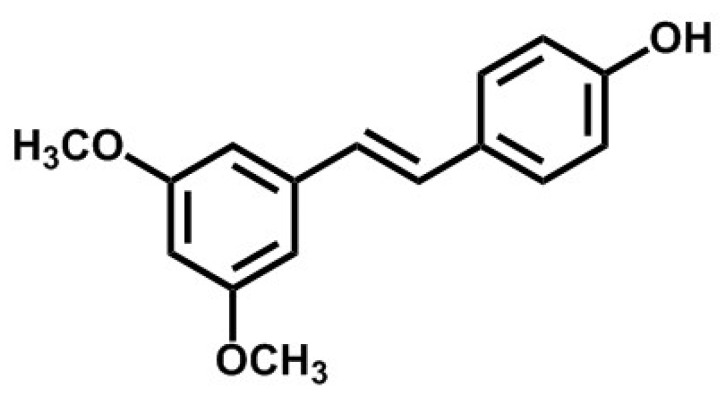
Chemical structure of pterostilbene.

An outstanding drug should have excellent bioactivities and appropriate pharmacokinetic parameters. Pterostilbene is characterized by low molecular weight (Figure 1) and good liposolubility, allowing it to easily cross the blood-brain barrier [16]. Experiments showed that pterostilbene can be rapidly absorbed and is widely distributed in the body. It has suitable metabolic stability and bioavailability [17,18], indicating its suitable pharmacokinetic characteristics. In addition, most available data in human and animal models show that pterostilbene has no significant toxic effects [19,20]. Therefore, pterostilbene is a potential natural small-molecular medicine with a good development prospect. This article reviews the progress in pterostilbene synthesis, bioactivities, and partial mechanisms and certain drug metabolism characteristics in vitro and in vivo. This review provides advantageous information for the comprehensive study of the pharmacokinetic features and the mechanisms underlying the bioactivity of pterostilbene.

## 2. Resources and Synthesis

### 2.1. Resources

Pterostilbene has a wide range of natural sources, such as *Dalbergia* and *Vaccinium* spp. Devaiah et al. collected berries in 2013, 2014, and 2015 from 42 grape varieties and assessed the ability of each grape variety to continuously produce four major stilbene compounds, namely, t-piceid, t-resveratrol, ε-viniferins, and t-pterostilbene. Pterostilbene widely existed in the different grape varieties [21]. Rimando et al. found pterostilbene in two *Vaccinium* (*V. ashei*) varieties, and its content in the dried sample was 99–520 ng/g [22].

### 2.2. Chemical Synthesis and Biosynthesis of Pterostilbene

In addition to being extracted from natural products, pterostilbene can also be synthesized by biological and chemical methods. The chemical synthesis of pterostilbene involves the following methods.

**Scheme 1:**
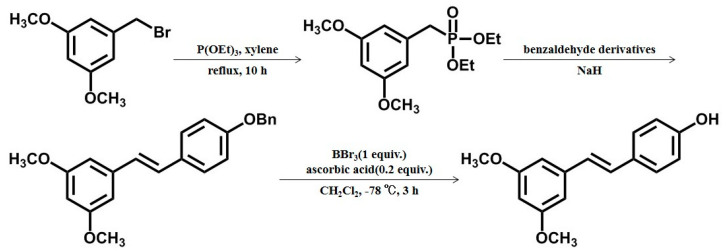
The first chemical synthesis method of pterostilbene [23].

**Scheme 2:**
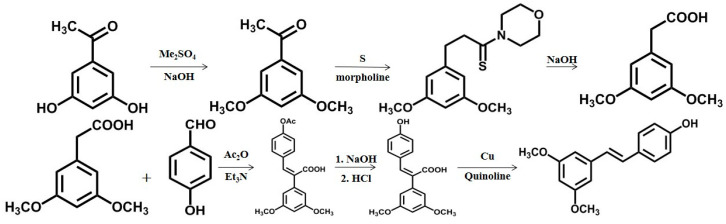
The second chemical synthesis method of pterostilbene [24].

**Scheme 3:**
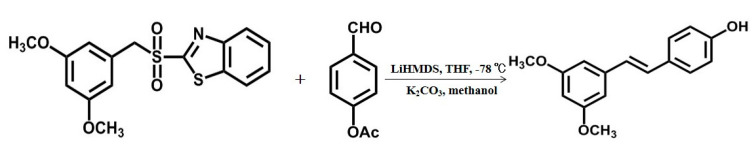
The third chemical synthesis method of pterostilbene [25].

**Scheme 4:**
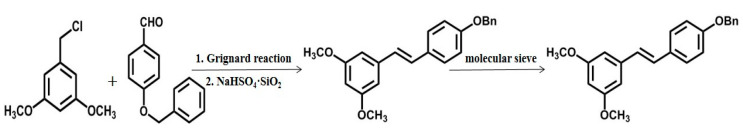
The fourth chemical synthesis method of pterostilbene [26].

(1) Pterostilbene was synthesized from 3,5-disubstituted benzyl bromide by Arbuzov, Wittig–Horner reaction and deprotection. Among them, boron tribromide and ascorbic acid were used in the process of deprotection, and the use of ascorbic acid can reduce the occurrence of polymerization in the process of deprotection (Scheme 1) [23]. This method has some shortcomings, such as purification and separation difficulties, high environmental pollution, and low synthesis yield.

(2) Pterostilbene was obtained from 3,5-dihydroxyacetophenone by methylation, rearrangement, perkin reaction, and decarboxylation-isomerization reaction (Scheme 2) [24]. Impurities were formed, and the yield was low due to the high decarboxylation temperature.

(3) Pterostilbene was synthesized through the Julia olefin reaction of 3,5-dimethoxybenzyl sulfone derivative and 4-acetoxy benzaldehyde under the action of hexamethyldisilylamino lithium (Scheme 3) [25], its further industrial application was limited by low temperature conditions.

(4) The trans stilbene skeleton was prepared by Grignard reaction and solid acid catalytic dehydration. The yield is approximately 77%, but controlling the anhydrous condition of grignard reagent in industrial scale-up production is an urgent problem (Scheme 4) [26].

**Scheme 5:**
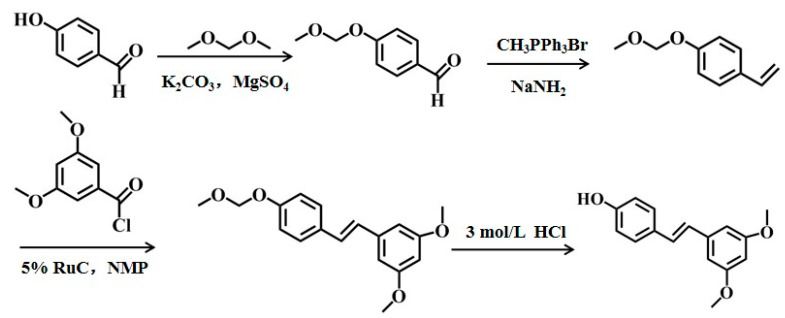
The fifth chemical synthesis method of pterostilbene [27].

Shi et al. [27] studied a new process (Scheme 5). The yield of the process was approximately 78%, the reaction conditions were mild, the market supply of raw materials was sufficient, and no special low temperature or pressure device was used. The whole process did not need column separation or distillation, and the product was purified only by recrystallization, which was suitable for industrial production.

Biosynthesis plays an important role in the synthesis of pterostilbene because of its high yield and low production cost. Resveratrol *O*-methyltransferase and grapevine stilbene synthetase can catalyze the resveratrol biosynthesis of pterostilbene in tobacco by agricultural osmosis technique; resveratrol *O*-methyltransferase gene expression in grape leaves was induced by different stress treatments [28]. First, Heo et al. first used an engineered strain to increase l-tyrosine, the initial precursor of pterostilbene. Second, they tried to use a medium containing l-tyrosine to produce pterostilbene in engineered *Escherichia coli* and obtained up to 33.6 ± 4.1 mg/L of pterostilbene in a minimal medium containing 1 mM l-methionine, which was about 3.6 times higher compared with that obtained from the parental *E. coli* strain harboring a plasmid for pterostilbene biosynthesis [29,30]. In the experiment of Kallscheuer et al., the synthesis of MalEEc-OMTVv fusion protein enabled the constructed *Corynebacterium glutamicum* strain to produce pterostilbene with a titer of 42 mg/L (0.16 mM) after 6 days of culture, thereby proving that *C. glutamicum* was suitable for the microbial production of pterostilbene [31].

## 3. Pharmacokinetics

Pterostilbene has suitable pharmacokinetic characteristics and no significant toxic effects. Although many biological similarities exist between pterostilbene and resveratrol, pterostilbene shows better bioactivity and bioavailability.

### 3.1. Absorption

Pterostilbene has more pharmacokinetic advantages than resveratrol. For instance, it can be rapidly absorbed. A pharmacokinetic study showed that at a single oral dose (pterostilbene 56 mg/kg, resveratrol 50 mg/kg), the peak plasma concentration (Cmax) values of pterostilbene were 36 times higher than those of resveratrol, the time to maximum plasma concentration (Tmax) value of pterostilbene was twice as fast as resveratrol The oral bioavailability of pterostilbene was 66.9%, whereas that of resveratrol was 29.8% [17]. The area under the curve (AUC) values of pterostilbene in Caco-2, HT29, and HCT116 cells were 2.6, 4.1, and 2.2 times higher than those of resveratrol, respectively [14,32,33]. From the perspective of chemical structure, pterostilbene has good bioavailability, probably owing to the presence of two additional methoxy groups on the A-benzene ring of resveratrol. These two methoxyl groups are responsible for its high lipophilicity, which may increase the permeability of the cell membrane and pterostilbene’s oral absorption. Experiments using Caco-2 cells as an in vitro model of human intestinal absorption showed that the absorption of pterostilbene prodrug containing isoleucine is due to passive diffusion and the expressions of H^+^-dependent transporters, such as PepT1 and OATP, on the apical membrane of intestinal cells [34]. Pterostilbene can be absorbed into the systemic circulation through the oral mucosa, thus, mixing pterostilbene into chewing gum or lozenges can be an innovative strategy to prevent oral cancer [34].

One of the main factors affecting the absorption of pterostilbene is the poor solubility (about 21 μg/mL). Different methods have been used to improve the water solubility of the compound. (1) When pterostilbene was co-crystallized with piperazine at a stoichiometric molar ratio of 2:1, its water solubility increased six-fold [1]. (2) The bioavailability of pterostilbene also improved by solubilized excipients such as 2-hydroxypropyl-β-cyclodextrin (HP-β-CD). The bioavailability of HP-β-CD pterostilbene solution (F = 59.2 ± 19.6%) was better than that of pterostilbene suspension (F = 15.9 ± 7.8%). The use of pterostilbene–cyclodextrin complex can slow down the rapid metabolism and elimination of pterostilbene, thereby improving its bioavailability [35]. (3) Fasting also affects the absorption of pterostilbene. Food accelerates bile secretion, which increases the water solubility of common compounds. Thus, co-administration of pterostilbene before or after meals can significantly maximize its oral absorption. Under the same dosing route and dose, the bioavailability of the free eating group was three times higher than that of the fasting group. Therefore, the differences in oral pharmacokinetics between suspension and solution and between fasting and non-fasting states were due to the difference in solubility [35]. (4) The modification of pterostilbene into a prodrug containing hydrophobic side chain amino acids can greatly promote its absorption. The hydroxyl group of pterostilbene was reversibly connected to the N-terminal carbamate of natural amino acids, which were easily absorbed by rats after intragastric administration. When using isoleucine or balanine prodrugs, the obtained AUC values of pterostilbene in the blood were highest. Compared with pterostilbene, the administration of isoleucine prodrug increased absorption, decreased metabolism, and maintained high concentrations for several hours in most of the examined organs [34]. The improvement of water solubility promotes the research development of pterostilbene.

### 3.2. Distribution

Pterostilbene had low molecular weight and good liposolubility, and its distribution volume (5.3 L/kg) was larger than that of whole body water (~0.7 L/kg). Li et al. specifically revealed the tissue distribution of pterostilbene in C57 BL/6 mice [16]. The tissue concentrations at 20 min after oral administration of 28 mg/kg pterostilbene were in the following order: stomach > liver > testis > kidney > intestine > lung > brain > spleen > skeletal muscle > heart. For most tissues, the content of pterostilbene decreased continuously with progressing sampling point (20–90 min), but the highest content of pterostilbene in the brain was 10.3 ± 3.2 μg/g at 45 min, indicating that pterostilbene may easily pass through the blood-brain barrier. Pterostilbene easily crosses the blood-brain barrier because of its good liposolubility. Therefore, in lipid-rich brain tissue, pterostilbene has a higher blood-brain partition coefficient than its sulfated metabolites. The pterostilbene content of some tissues was much higher than that of in the blood [36], which elucidates why pterostilbene can be bioactive even at low blood or plasma concentrations. The above mentioned research shows that pterostilbene is widely distributed in various tissues and may be highly distributed in some organs.

### 3.3. Metabolism

Phase II metabolism was the main metabolic type of pterostilbene. In male Wistar rats, sulfate was the main metabolite, and only a small amount of glucuronic acid was found in the liver [36]. In mice, glucuronide and sulfate metabolites were the main metabolites of pterostilbene [37]. As determined by human liver microsomes under the same conditions, 68% of resveratrol was bound to glucuronic acid, more than 75% of pterostilbene remained unchanged, and only 4′-OH could be used for sulfation [38]. Therefore, pterostilbene has high metabolic stability and bioavailability in the human body. However, due to the significant differences in species, dose, and other factors, the metabolic process may also significantly differ.

Intestinal microorganisms may demethylate pterostilbene in CD-1 mouse. However, further studies are needed to confirm this hypothesis [39]. Some studies have shown that pterostilbene significantly inhibits the activities of CYP2C8, UGT1A9, and UGT1A6 in vitro and may inhibit metabolism through these enzymes in vivo [40,41]. Human UGT1A1 and 1A3 were identified as the two major UGTs responsible for the glucuronic acid oxidation of pterostilbene [34,39]. Moreover, Dellinger et al. found the possible existence of gender differences in the metabolism of pterostilbene, which may be due to the difference in the expression of UGT1A1 between sexes [38]. Clinical studies are necessary to evaluate the correlation of these interactions in vivo.

### 3.4. Excretion

Pterostilbene is excreted in the form of glucuronic acid in male Sprague-Dawley rats. Most of the glucuronic acid-binding metabolites were excreted 12 h after administration, whereas the amount of pterostilbene excreted in urine increased steadily at 120 h after administration. Glucuronic acid-bound pterostilbene metabolites increased at 2 h, indicating their hepatointestinal circulation. These metabolites were eliminated through non-renal pathways, and renal excretion and hepatic excretion accounted for about 0.219% and 99.78% of the total excretion, respectively [42].

At high doses, pterostilbene showed limited elimination ability, because the binding enzyme may be saturated or partially saturated. Pterostilbene showed a lower clearance rate than resveratrol and therefore showed longer therapeutic time window according to Yeo et al. [1] This finding can well prove the structural difference between the two compounds; pterostilbene was less sensitive to binding metabolism. Samuel et al. [35] studied the differences in the clearance rate of pterostilbene at different doses. The clearance rate was 68.2 ± 9.8 mL/min/kg at 2.5 mg/kg and 36.4 ± 7.8 mL/min/kg at 25 mg/kg (Table 1). The clearance rate decreased nearly twice, and the increase of dose led to the decrease of scavenging rate, which may be due to the metabolic saturation or partial saturation of pterostilbene. The limited capacity for pterostilbene elimination prompts researchers to choose doses cautiously in future studies, because even moderate changes in drug metabolic capacity between patients or subjects can lead to significant differences in systemic drug exposure and treatment responses

## 4. Bioactivities of Pterostilbene

**Figure 2:**
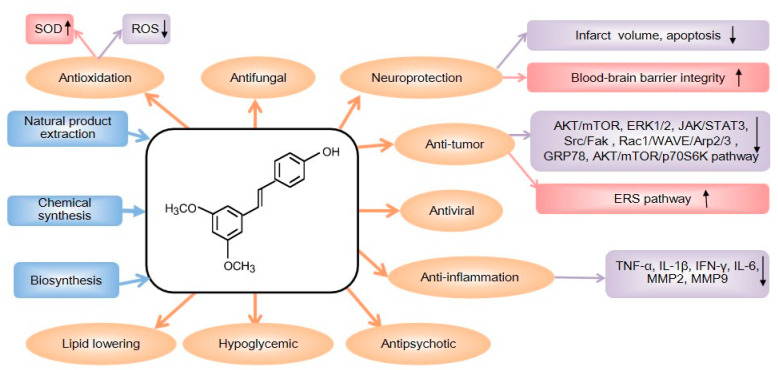
Bioactivities and possible mechanisms of pterostilbene. The anti-tumor mechanism of pterostilbene is related to several tumor characteristics, including anti-proliferation (AKT/mTOR, ERK1/2 and JAK/STAT3 pathway), the induction of apoptosis (AKT/mTOR/p70S6K and ERS pathway), the inhibition of invasion and metastasis (Src/Fak and Rac1/WAVE/Arp2/3 pathway), and the inhibition of tumor stem cells (GRP78 pathway). AKT, active human protein kinase; mTOR, mammalian target of rapamycin; ERK1/2, extracellular signal-regulated kinase; JAK/STAT3, Janus kinase/signal transducer and activator of transcription; p70S6K, downstream of mTOR; ERS, endoplasmic reticulum stress; Src/Fak, steroid receptor coactivator/focal adhesion kinase; Rac1, Rac family small GTPase 1; WAVE, WASP-family verprolin-homologous protein; Arp2/3, actin-related protein 2/3; GRP78, glucose-regulated protein; SOD, superoxide dismutase; ROS, reactive oxygen species; TNF-α, tumor necrosis factor-α; IL-1β, interleukin-1β; IFN-γ, interferon-γ; IL-6, interleukin-6; MMP2, matrix metalloprotein-2; MMP9, matrix metalloprotein-9.

Pterostilbene exerts its bioactivities through a variety of mechanisms. Pterostilbene exerts its anti-tumor effect by regulating a variety of signal pathways and plays a neuroprotective role by improving and reducing the volume of cerebral infarction, inhibiting apoptosis, and protecting the integrity of the blood-brain barrier. Antioxidant and anti-inflammatory activities serve as the basis for the various bioactivities of pterostilbene (Figure 2). In addition, pterostilbene shows hypoglycemic, lipid-lowering, antifungal, antiviral, and antipsychotic activities. An in-depth understanding of the bioactivities and mechanisms can help determine the potential therapeutic application of pterostilbene.

### 4.1. Anti-Tumor Activity

Anti-tumor activity is the focus of its research. In vitro, pterostilbene inhibited the proliferation of a variety of tumor cells, including stomach, lung, liver, oral cavity, pancreas, lymph, colon, prostate, breast, melanoma, leukemia, and myeloma tumor cells. In vivo, pterostilbene inhibited tumor occurrence and metastasis and showed almost no toxicity [44,45]. Manlio et al. found that pterostilbene had a stronger ability to induce the apoptosis of chronic myeloid leukemia cells than resveratrol. Resveratrol has almost no effect on inducing apoptosis of drug-resistant cancer cell lines, while pterostilbene can induce apoptosis of drug-resistant cancer cell lines, and there was no significant difference between the AC_50_ of drug-resistant cancer cell lines and the AC_50_ of sensitive cancer cell lines, indicating that pterostilbene has a strong apoptosis-inducing effect on drug-resistant cancer cell lines. Pterostilbene was less toxic on normal hemopoietic stem cells than on leukemia cells [15]. Cultured human glioblastoma cell lines U87MG and GBM8401, human promyelocytic acute leukemia cell line HL-60, gastric cancer AGS cells, colon cancer COLO205 cells, colorectal cancer HT-29 cells, and hepatocellular carcinoma HepG2 cells were reduced in a time- and concentration-dependent manner, with IC_50_ values of 1.42, 2.99, 46.7, 50.7, 71.2, and 82.8 μM respectively [46,47]. The signal pathways related to its mechanism are summarized in Table 2.

Sun et al. [62] determined for the first time that pinostilbene was the main pterostilbene metabolite in the colon of pterostilbene-fed mice. The inhibitory effect of pinostilbene on the growth of human colon cancer cells was similar to that of pterostilbene, and these effects were related to the regulation of a variety of key proteins involved in cell cycle arrest and apoptosis. The bioactivities of pinostilbene in the colon and its significance in the prevention of colon cancer by oral food containing pterostilbene need to be studied to further clarify the mechanism of pterostilbene’s inhibition of the growth of colon cancer cells. This research also inspired us to combine the bioactive metabolites of pterostilbene with the bioactivities of pterostilbene in a follow-up study to further clarify the biological mechanism of pterostilbene.

There are also some novel anti-tumor effects such as autophagy or apoptosis-independent pathways of pterostilbene. Ko et al. studied the effect of pterostilbene on human oral cancer cells and found that pterostilbene can induce autophagy of oral cancer cells by activating JNK1/2, inhibiting Akt, ERK1/2 and p38, which is expected to be a new drug for the treatment of oral cancer [63]. Wang et al. depleted PolyFN suspended Lewis lung cancer cells by silencing endogenous FN expression or pterostilbene, and observed whether pterostilbene could inhibit the metastasis of lung cancer cells. It was found that pterostilbene inhibited PolyFN assembly and lung metastasis on Lewis lung cancer suspension cells regulated by AKT/ERK. Its preventive and therapeutic effects on lung metastasis of lung cancer cells include apoptosis-independent manner [64].

### 4.2. Anti-Inflammation Activity

Anti-inflammatory effect is another important research field in the study of pterostilbene’s bioactivities.

In vitro experiments showed that pterostilbene could significantly reduce the expression of pro-inflammatory mediators TNF-α, IL-1β, IL-6, matrix metallopeptidase 2 (MMP2), and matrix metallopeptidase 9 (MMP9) in hypertonic cultured human corneal epithelial cells and had a protective effect on human corneal inflammation induced by hyperosmotic stress [65]. Pterostilbene significantly reduced inflammatory reaction by reducing the production of cytokines sICAM1, IL-8, MCP-1, and sE-selectin and by inhibiting the adhesion of U937 monocytes to human umbilical vein endothelial cells. Furthermore, pterostilbene decreased the expression of ERS-related proteins eIF2a, ICAM1, MMP9, and GRP78 under endoplasmic reticulum stress, which effectively reduced the inflammation of vascular endothelial cells [9]. Pterostilbene (IC_50_, 22.4 μmol/L) was more effective as an anti-inflammatory compound than resveratrol (IC_50_, 43.8 μmol/L) in inhibiting the occurrence of colon cancer in HT-29 human adenocarcinoma cell line [16].

In vivo, pterostilbene showed its potential anti-inflammatory effect by inhibiting the lipopolysaccharide-induced expressions of IL-6 and TNF-α mRNA in rat hippocampus. The decrease of inflammatory cytokines (IL-1β, TNF-α, and IFN-γ) in streptozotocin (STZ)-induced diabetic mice proved that treatment with pterostilbene can significantly improve inflammatory response [66]. In addition, pterostilbene may become a new therapeutic intervention to reverse vascular inflammation by inhibiting the metabolic activity of intestinal microflora and reducing the metabolism of carnitine into trimethylamine-N-oxide [67]. What is more, targeting to NLRP3 is a novel anti-inflammation effect of pterostilbene. Liu et al. analyzed the inflammasome activity of nucleotide-binding oligomerization domain-like receptor family pyrin domain-containing 3 (NLRP3) by Western blot, and found that pterostilbene treatment decreased the activation of NLRP3 inflammasome, suggesting that pterostilbene may reduce early brain injury after subarachnoid hemorrhage by inhibiting NLRP3 inflammatory bodies [68]. These results suggest that pterostilbene has potential as an anti-inflammatory agent.

### 4.3. Neuroprotective Activity

#### 4.3.1. Cerebral Ischemia

Pterostilbene plays an important role in the occurrence and development of cerebral ischemia. Zhou et al. [69] found that after oral administration, pterostilbene improved motor function in a time- and concentration-dependent manner before and after ischemia. It reduced the infarct volume and weakened the damage to the blood-brain barrier after ischemia reperfusion in the mouse model of middle cerebral artery occlusion. The best dose was 10 mg/kg, which was administered within 1 h after ischemia reperfusion. This protective effect may be at least partly related to the inhibition of oxidative stress and apoptosis of neurons in the penumbra of the cortex. Another article showed the same conclusion by studying this model; however, the mechanism was pterostilbene’s reduction of astrocyte-mediated inflammation and oxidative damage after ischemia reperfusion by inhibiting NF-κB phosphorylation and nuclear translocation [70].

Pterostilbene was used to treat mouse brain in a common carotid artery occlusion model in vivo and HT22 neuronal cells in vitro. Pterostilbene reduced mitochondrial oxidative damage induced by cerebral ischemia-reperfusion by activating heme oxygenase-1 (HO-1) signal pathway [71]. Longxue Tongluo capsule was widely used to treat ischemic stroke. Jing et al. quantified the multi-components in the plasma or tissue of rats after oral administration of Longxue Tongluo capsule and clarified the kinetic characteristics of the main phenolic derivatives. Among the 11 analytes, the concentration of pterostilbene in the brain was the highest (141.4 ± 25.48 ng/g) [72]. These findings indicated that pterostilbene may be effective for alleviating neurological and histological abnormalities after cerebral ischemia reperfusion.

#### 4.3.2. Alzheimer’s Disease

Pterostilbene can be used to treat Alzheimer’s disease (AD). Chang et al. found that pterostilbene was a more effective cognitive and cellular stress regulator than resveratrol in mouse susceptibility No. 8 (SAMP8) AD model of accelerated aging at the same and achievable dietary dose of 120 mg/kg. This action may be due to the increase of liposolubility caused by the substitution of hydroxyl groups in pterostilbene, which increased the expression of peroxisome proliferator-activated receptor α [10]. Pterostilbene can alleviate lipopolysaccharide-induced learning and memory impairment. The underlying mechanism may be related to its inhibition of microglial activation, which resulted in the obvious decrease of LPS-induced production of NO, TNF-α, and IL-6 in N9 microglial cells and the protection of neuronal damage. Thus, pterostilbene improved LPS-induced learning and memory impairment by suppressing doublecortin expression and increasing neuronal nuclear antigen expression [73]. Moreover, pterostilbene inhibited Aβ apoptosis through PI3K/Akt signal pathway [74] through its antioxidant activity in the brain and further through the inhibition of acetylcholinesterase activity; thus, it had anti-AD activity [75]. These results suggest that pterostilbene may be involved in the dietary intervention for AD prevention and may be used as an adjuvant treatment of AD.

#### 4.3.3. Others

Pterostilbene attenuated glutamate-induced oxidative stress damage of HT22 cells in mouse hippocampal neurons through the Nrf2 signal pathway [76] and attenuated the central nervous system damage induced by high glucose via the activation of nuclear factor erythroid 2-related factor 2 [77]. Pterostilbene preconditioning can improve tissue and functional damage through oxidative stress, programmed cell death, and inflammation mediated by HO-1, thereby preventing neonatal brain injury caused by hypoxic-ischemic encephalopathy [78]. Pterostilbene showed estrogenic activity in its neuroprotection effect by up-regulating the expression of anti-apoptotic Bcl-2 and activating MAPK/ERK and PI3K/AKT signal pathways. ER-α was the main receptor involved in the neuroprotective effect of pterostilbene [79]. Therefore, pterostilbene may be a putative neuroprotective substance for nerve injury treatment and prevention.

### 4.4. Antioxidation Activity

The anti-oxidation effect of pterostilbene is the basis for its use in the treatment of numerous diseases. In vitro, nuclear transcription factor (Nrf2) is the “main regulator” of cytoprotective and antioxidant genes. In the STZ-induced diabetic model (Table 3), Nrf2 activation and its downstream target gene expression were observed during pterostilbene treatment, the oxidative damage on the pancreatic tissue was reduced [69]. As an effective activator of Nrf2, pterostilbene protected against arsenic-induced cytotoxicity and apoptosis of human keratinocytes [80]. In a high glucose environment, the proliferation of human retinal endothelial cells was enhanced, the expressions of TNF-α and IL-1β increased, the level of NF-κB protein was significantly up-regulated, the production of reactive oxygen species (ROS) significantly increased, and the activity of superoxide dismutase (SOD) significantly decreased. Compared with the high glucose group, pterostilbene significantly inhibited the excessive proliferation of human retinal endothelial cells, decreased TNF-α and IL-1β levels, inhibited the expression of NF-κB protein, decreased the production of ROS, and increased SOD activity. Pterostilbene may inhibit the excessive proliferation of human retinal endothelial cells (hRECs) through antioxidation, thereby delaying the progression of diabetic retinopathy [81]. In vivo, rats were administered 50 ppm pterostilbene daily for 6 weeks. By activating the Nrf2 signal pathway, the expressions of HO-1 and glutathione reductase were significantly increased, and antioxidation ensued. Cellular defense mechanisms, such as the activities of antioxidant enzymes (SOD, catalase, and glutathione peroxidase), protected cells from active free radicals and other oxidants. The activities of SOD, catalase, glutathione peroxidase, and glutathione transferase and glutathione content in the liver and kidney of diabetic rats were significantly lower in the treatment group than in the normal control group. After treatment with 40 mg/kg pterostilbene for 6 weeks, the abovementioned activities significantly improved [8]. These results indicate that pterostilbene is a promising antioxidant.

### 4.5. Lipid-Lowering Activity

#### 4.5.1. Serum Lipids

A study by Rimando et al. stated that pterostilbene is an agonist of peroxisome proliferator-activated receptor; it had the same activity as the fibrate antihyperlipidemic drugs used in clinic [11]. Resveratrol is not an activator of peroxisome proliferator-activated receptor. Compared with resveratrol, pterostilbene may be a more effective hypolipidemic compound, making it a possible choice for the treatment of dyslipidemia [11].

#### 4.5.2. Liver Steatosis

Pterostilbene exerts its lipid-lowering activity by reducing the amount of white adipose tissue and increasing the amount of brown adipose tissue. Koji et al. [82] found that pterostilbene inhibits the accumulation of white adipose tissue by enhancing energy metabolism and by partially inhibiting adipogenesis in obese Otsuka Long–Evans Tokushima fatty rats (Table 4). Pterostilbene can also induce the browning of white adipose tissue. The analysis of inguinal white adipose tissue showed that the tendency of browning and the transcription of several marker genes (CIDA, EBF2, PGC1α, PPARγ, Sirt1, and Tbx1) increased significantly [83]. Aguirre et al. studied brown adipose tissue. By analyzing the effects of two doses of pterostilbene (15 and 30 mg/kg/d) on several indexes of thermogenic ability in a hereditary obesity model, researchers found that pterostilbene could improve the thermogenic and oxidative capacities of brown adipose tissue in obese rats [84]. In addition, the degreasing mechanism of pterostilbene was determined. Pterostilbene decreased fatty acid availability and promoted the synthesis of triglyceride, the assembly of very low density lipoprotein, and the oxidation of fatty acids [85].

#### 4.5.3. Obesity

Under overfeeding conditions, pterostilbene can reduce the weight of subcutaneous adipose tissue and prevent the decrease of triglyceride 9 caused by tumorigenic feeding, thereby reducing the absorption of glycerol and promoting the accumulation of triglycerides [86]. Gómez-Zorita et al. studied the effects of pterostilbene on rats fed an obesogenic diet (Table 4). The decrease of lipogenesis in adipose tissue and the increase of fatty acid oxidation in liver contributed to the anti-obesity effect of pterostilbene. If we compare this result with that of resveratrol, then we would observe that pterostilbene is more effective than resveratrol at a dose of 15 mg/kg/d [87]. Gómez-Zorita et al. also observed that pterostilbene had an anti-adipogenic effect on preadipocytes and quickly inhibited the incorporation of glucose into the lipids of mature adipocytes, this anti-lipid effect can occur in vivo [88]. Studies by Chin et al. showed that pterostilbene had an anti-adipogenic effect on preadipocytes, differentiated adipocytes, and mature adipocytes [89]. Pterostilbene can also change the composition of intestinal microflora. The weight loss and cholesterol-lowering effect induced by pterostilbene in Zucker rats may be related to the enrichment of the Verrucomicrobia phylum [90]. Pterostilbene can potentially be used to treat and prevent obesity due to its lipid-lowering activity.

### 4.6. Hypoglycemic Activity

Pterostilbene exerts its hypoglycemic activity by stimulating the increase of glycolysis and the decrease of gluconeogenesis. In STZ-nicotinamide-induced diabetic male albino Wistar rats (Table 5), the concentrations of blood glucose and glycosylated hemoglobin were significantly decreased after oral administration of pterostilbene (10, 20, and 40 mg/kg) for 2, 4, and 6 weeks. After treatment, the expressions of hexokinase increased significantly, whereas the expressions of glucose-6-phosphatase, and fructose-1,6-bisphosphatase, decreased significantly. The expression of glucose-6-phosphatase and fructose-1-line 6-diphosphatase regulated glucose homeostasis [91].

Nuclear transcription factor Nrf2 is the “main regulator” of cytoprotective and antioxidant genes. Bhakkiyalakshmi et al. evaluated the effect of pterostilbene on islet β cytotoxicity in INS-1E rats induced by STZ. The cells were pretreated with pterostilbene in a time- and concentration-dependent manner (0–48 h and 0–16 μM, respectively) and then treated with 10 mM STZ for 1 h. Pterostilbene pretreatment (0–8 μM) for 48 h significantly protected islet β-cell from damage induced by STZ in a time-dependent manner. The survival rate of pretreated cells at concentrations of 4 and 8 μM increased by 67 ± 3.4% and 72 ± 2.7%, respectively. The activation of Nrf2 and its downstream target gene expression was observed during pterostilbene treatment, which played a protective role in islet β cells [12]. This finding showed that pterostilbene can exert its hypoglycemic activity by protecting the islet β cells. Elango et al. injected several small doses of STZ (50 mg/kg) intraperitoneally into 6-week-old male albino Swiss mice for 5 days consecutively. After 1 week, moderate diabetic mice with glycosuria and hyperglycemia (with blood glucose concentrations of 200 and 300 mg/d) were injected intraperitoneally with pterostilbene (5 mg/kg), resveratrol (10 mg/kg), and glibenclamide (600 μg/kg body weight) for 5 weeks. The mice with hyperglycemia were intraperitoneally injected with pterostilbene (10 min), resveratrol (10 min), and glibenclamide (600 μg/kg body weight) for 5 weeks. Compared with resveratrol, a known Nrf2 activator, pterostilbene administration significantly improved the level of serum insulin in diabetic mice. The ability to reduce the level of fasting blood glucose in diabetic mice may be due to the increased insulin secretion of residual β-cells, which is equivalent to the activity of the antidiabetic drug glibenclamide. Compared with diabetic mice, those treated with pterostilbene showed restored activity of downstream targets of Nrf2; pterostilbene further limited the production of free radicals [92]. Pterostilbene plays a hypoglycemic role by stimulating insulin secretion of the residual islet β cells.

These are the effects of pterostilbene on type 2 diabetic rats induced by STZ-nicotinamide. Gómez-Zorita et al. studied the effect of pterostilbene on insulin resistance associated with obesity feeding. Wistar rats were randomly divided into three experimental groups with nine rats in each group, which were fed with a commercial obesogenic diet that was high in sucrose (20.0%) and fat (22.5%). The latter two groups were added to the diet according to the guaranteed amount of 15 mg/kg body weight/d or 30 mg/kg body weight/d. The duration of the experiment was 6 weeks. The results showed that pterostilbene improved the serum glucose control of insulin-resistant rats induced by obese diet. The mechanism may be the increase of liver glucokinase activity and skeletal muscle glucose uptake. Compared with their previously reported results on the effects of resveratrol, the dose of 15 mg/kg body weight/d was not as effective as pterostilbene in reducing serum glucose levels [93].

Pterostilbene exerted its hypoglycemic activity by inhibiting the apoptosis of islet β cells. It increased the expression of anti-apoptotic protein Bcl-2 and down-regulated the expression of pro-apoptotic Bax and caspase-3, thereby inhibiting the apoptosis of islet β cells in diabetic rats [14]. A recent study suggested that the anti-diabetic mechanism of pterostilbene may be related to the activation of PPARγ and PI3K/AKT signaling pathways in adipose tissue [94]. These results suggest that pterostilbene may be a candidate for the treatment of diabetes in the future.

### 4.7. Others

#### 4.7.1. Antifungal Activity

Studies on antifungal activity showed that 160 μg/mL of resveratrol and 60 μg/mL (Table 6) of pterostilbene completely inhibited the germination of conidia of *Botrytis cinerea* [2]. In addition, pterostilbene had a strong bacteriostatic effect on *Fusobacterium nucleatum*, a key periodontal pathogen, and the minimum inhibitory concentration was more than 60 times lower than that of resveratrol; pterostilbene’s antibacterial activity mechanism involved inducing the leakage of cell contents, which resulted in the loss of bacterial cell vitality [95]. Pterostilbene is expected to be a candidate for adjuvant treatment of periodontitis.

#### 4.7.2. Antiviral Activity

Resveratrol and pterostilbene completely blocked HIV-1 infection in resting CD4 T cells in the reverse transcription step at low molar concentrations. Resveratrol alone did not inhibit HIV-1 infection in activated T cells, but it co-inhibited reverse transcription with nucleoside reverse transcriptase inhibitors in these cells [96]. In the transformed fibroblast cell line (293T), 30 μM resveratrol had no anti-HIV-1 effect, whereas 10 μM pterostilbene showed 50% inhibitory effect [97]. Thus, resveratrol and pterostilbene are promising adjuvants in anti-HIV pre-exposure prophylaxis formulations.

#### 4.7.3. Antipsychotic Activity

Pterostilbene showed anti-anxiety effect by down-regulating the phosphorylation level of extracellular regulated kinase in the hippocampus of mice [98]. Pterostilbene had a similar antidepressant effect on chronic unexpectedly stressed rats (Table 6), and its mechanism may involve the promotion of hippocampal neurogenesis; its molecular mechanism may be related to the activation of BDNF/ERK/CREB neurotrophic signal pathway [99]. Pterostilbene’s antipsychotic activity warrants further study.

## 5. Conclusions and Prospects

Pterostilbene is available from a wide range of natural sources. Besides extraction from natural products, pterostilbene can be obtained by biosynthesis and chemical synthesis. This paper reviews the research progress in the biosynthesis of pterostilbene by using genetic engineering technology to construct engineered bacteria and realize the synthesis of l-tyrosine.

In terms of neuroprotective effect, because of its low molecular weight and good liposolubility, pterostilbene can easily pass through the blood-brain barrier, laying the foundation for its medicinal properties. It can improve neurological function and alleviate histological abnormality after cerebral ischemia-reperfusion. It can inhibit the apoptosis of Aβ through PI3K/Akt signal pathway and further inhibit the activity of acetylcholinesterase, thereby showing anti-AD activity. The anti-tumor effect of pterostilbene is accurate and has been widely studied. In vitro, pterostilbene inhibits the proliferation of many kinds of tumor cells. In vivo, pterostilbene inhibits tumor occurrence and metastasis. It is more effective than resveratrol in inducing the apoptosis of chronic myeloid leukemia cells, and it may be widely used as an antineoplastic drug in the future. Pterostilbene exhibits an anti-inflammatory effect by reducing the release of pro-inflammatory mediators. Pterostilbene can also activate Nrf2 signal pathway, thereby acting as an antioxidant. In the process of emergence and development, many diseases are often related to inflammation and oxidative damage. Pterostilbene has significant antioxidant and anti-inflammatory activities, indicating its potential for use in the treatment of various diseases. As an anti-inflammatory compound, pterostilbene is more effective than resveratrol in inhibiting the occurrence of colon cancer in HT-29 human adenocarcinoma cell line. The anti-inflammatory effect of pterostilbene has a broad prospect and needs further study. The specific comparison between pterostilbene and resveratrol is shown in Table 7.

Pterostilbene is a promising candidate drug, which may enter human intervention trials. In terms of safety, pterostilbene has no significant toxic effects according to most of the data available in human and animal models from preclinical and clinical trials [19,20]. In terms of efficacy, pterostilbene has great potential as an anti-tumor drug, as indicated in many studies. Pterostilbene is the main and important component of Dragon’s blood. Dragon’s blood has been used as a renowned traditional medicine in various cultures around the world [3]. This plant has a long and extensive history of folk medicine in the treatment of injury, promotion of wound healing, and the treatment of cardiovascular and cerebrovascular diseases, which will possibly lead to its clinical breakthrough and clinical application fields and therapeutic features in various fields. In addition, pterostilbene has appropriate pharmacokinetic characteristics. Therefore, as a potential and promising anticancer and hypoxic-ischemic encephalopathy compound, it has attracted extensive attention in preclinical research. It is expected to become a candidate drug in clinical trials due to its efficacy and safety.

A new study [41] suggested that 100 mg/d or higher doses of pterostilbene may lead to at least a 50% increase in AUC from drugs cleared by UGT1A9, such as furosemide, mycophenolic acid, phenylbutazone, paracetamol, propofol, sulfinpyrazone, baicalein, quercetin, kaempferol, apigenin estrogens, and prostaglandins. The coadministration of pterostilbene with drugs primarily cleared by UGT1A9 might lead to potential drug interactions, and thus, preventive measures should be considered. Pterostilbene has a wide range of bioactivities. Network pharmacology and molecular target docking can be performed to explore its application in the treatment of other diseases.

## Figures and Tables

**Table 1:** Pharmacokinetics profiles of pterostilbene.

Subject	Mode of Administration	Dose (mg/kg)	AUC (mg h/L)	V (L/kg)	CL (mL/min/kg)	T_1/2_ (min)	Reference
C57 BL/6 mice	iv.	10	26.7 ± 8.2	Vc 0.674 ± 0.12	(0.014 ± 0.003) × 10^3^	34.5 ± 1.0	[16]
C57 BL/6 mice	ig.	14	4.43 ± 2.0	Vc 4.9 ± 1.9	(0.027 ± 0.008) × 10^3^	102± 19.2	[16]
C57 BL/6 mice	ig.	28	11.9 ± 2.1	Vc 3.76 ± 0.85	(0.012 ± 0.008) × 10^3^	87.8 ± 29.2	[16]
C57 BL/6 mice	ig.	56	39.4 ± 9.7	Vc 1.57 ± 0.27	(0.030 ± 0.005) × 10^3^	56.9 ± 13.9	[16]
SD rat	iv.	2.5	(35.6 ± 5.1)/60	Vc 2.85 ± 0.50	68.2 ± 9.8	93.9 ± 22.3	[35]
SD rat	iv.	5	(135,650 ± 8944)/(60 × 10^3^)	Vc (1267 ± 364)/10^3^	37.0 ± 2.5	96.6 ± 23.7	[43]
SD rat	iv.	10	(168.7 ± 28.6)60	Vc 3.03 ± 0.88	59.1 ± 8.8	155.1 ± 64.1	[35]
SD rat	iv.	11.2	4009/10^3^	Vss 5.30	2.7 × 10^3^/60	2.9 × 60	[17]
SD rat	iv.	20	17.5 ± 6.6	Vd 2.41 ± 1.13	(0.960 ± 0.025) × 10^3^/60	(1.73 ± 0.87) × 60	[39]
SD rat	iv.	25	(689.2 ± 124.1)/60	Vc 2.19 ± 0.17	36.4 ± 7.8	150.8 ± 15.9	[35]
Wistar rat	iv.	22.5	(38.8 ± 5.3) × 256.3/10^3^	Vss 6.1 ± 1.0	(2.3 ± 0.3) × 10^3^/60	(1.8 ± 0.3) × 60	[36]

CL, clearance; iv., intravenous; ig., intragastric; T_1/2_, half-life; Vd, volume of distribution; Vss, apparent distribution volume at steady concentration; Vc, apparent distribution volume of central compartment.

**Table 2:** Signaling pathway of the anti-tumor effect of pterostilbene.

Signaling Pathway	Model	Dose	Reference
EGFR, Akt/mTOR, Stat3, ERK1/2, and NFκB pathways	urethane-caused lung tumor	250 mg/kg	[48]
microRNA 448 circuit	MDA-MB-231 cells were cocultured with M2 TAM and were subcutaneously injected into the left flank of NOD/SCID mice	5 mg/kg	[49]
JAK/STAT3 signaling pathway	Breast cancer cell lines (MDA-231 and ZR-751)	75 μM	[50]
Src/Fak signaling pathway	MDA-MB-231-bearing NOD/SCID mice	10 mg/kg	[51]
Rac1/WAVE/Arp2/3 pathway	MDA-MB-231 cells	10 μM	[52]
β-catenin/p65 downstream signaling pathway	F344 rats were given two AOM injections subcutaneously	0.004% in the diet for 45 weeks	[53]
ATM/CHK/p53 pathway	non-small cell lung cancer cell (A549)	21 μM	[54]
GRP78 signaling pathway	human glioblastoma cell lines GBM8401 and U87MG	2.99, 1.42 μM	[46]
p53/SOD2/ROS pathway	HepG2 cells	100 μM	[55]
miR-663b/BCL2L14 signaling pathway	HTB-111 and Ishikawa cells	71.64 nM, 74.34 µM	[56]
JAK2/STAT3 signaling pathway	human osteosarcoma cell line, SOSP-9607	1.81 µM	[57]
AKT/mTOR/p70S6K and ERK1/2 pathways	T24 human bladder cancer cell	66.58 ± 1.84 µM	[58]
Fas/FasL pathway	human AGS gastric carcinoma cells (CCRC 60102)	50.7 μM	[47]
ERS signaling pathway	Human EC109 and TE1 esophageal cancer cells	150 μM	[59]
RAGE/PI3K/Akt signaling pathway	MIA PaCa-2 and MIA PaCa-2^GEMR^ cells (GEM-resistant cells)	41.8, 42.0 µM	[60]
signal transducer and activator of transcription 3 signaling pathway	HeLa, CaSki, and SiHa cervical cancer adherent cells	32.67, 14.83, 34.17 µM	[61]

**Table 3:** Model and dose of antioxidation activity of pterostilbene.

Model	Dose	Reference
Human keratinocytes, mouse epidermal cells	3.75, 7.5, 15 μM	[80]
human retinal endothelial cells	1.0 mM	[81]
Streptozotocin–nicotinamide-induced type 2 diabetes mellitus in Wistar rats	40 mg/kg for 6 weeks	[8]

**Table 4:** Model and dose of lipid-lowering activity of pterostilbene.

Model	Dose	Reference
H4IIEC3 cells	100 µM	[11]
Obese Otsuka Long–Evans Tokushima fatty rats	0.5% diet for 4 weeks	[82]
3T3-L1 mature adipocytes	5 µM	[83]
Mice fed an obesogenic high-fat diet	352 µmol/kg/d for 30 weeks	[83]
Genetic obesity Zucker (*fa/fa*) rats	15, 30 mg/kg body weight/day for 6 weeks	[84,85]
Wistar rats fed an obesogenic diet	15, 30 mg/kg body weight/day for 6 weeks	[86,87]
3T3-F442A preadipocytes	1–10 μM	[88]
3T3-L1 preadipocytes	5–40 μM	[89]
Genetic obesity Zucker (*fa/fa*) rats	15 mg/kg body weight/day for 6 weeks	[90]

**Table 5:** Model and dose of hypoglycemic activity of pterostilbene.

Model	Dose	Reference
Streptozotocin-nicotinamide-induced diabetic male albino Wistar rats	10, 20, 40 mg/kg for 2, 4, 6 weeks	[91]
Islet β cells of INS-1E rats induced by streptozotocin	4, 8 μM	[12]
Moderate diabetic mice with glycosuria and hyperglycemia	5 mg/kg for 5 weeks	[11]
Insulin resistance associated Wistar rats with obesity feeding	15 mg/kg body weight/d for 6 weeks	[93]
Diabetes was induced in rats by streptozotocin and a high-sugar and high-fat diet	20, 40 and 80 mg/kg/d for 8 weeks	[94]

**Table 6:** Model and dose of antifungal, antiviral and antipsychotic activity of pterostilbene.

Model	Dose	Reference
Conidia of *Botrytis cinerea*	60 μg/mL	[2]
*Fusobacterium nucleatum*	0.02 mg/mL	[95]
HIV-1 infection in resting CD4 T cells	5 μM	[96]
Transformed fibroblast cell line	10 μM	[97]
Elevated plus maze test mice	2 mg/kg	[98]
Chronic unexpected stressed model rats	2.5 mg/L	[99]

**Table 7:** Comparison between pterostilbene and resveratrol.

In Vivo or In Vitro	Model	Comparison	Mechanism	Reference
In vitro	Multidrug resistant HL60-R (human myeloid cell line expressing P-glycoprotein)	AC_50_ of pterostilbene is 85 ± 11, resveratrol has almost no effect on inducing apoptosis	Caspase-independent pathway	[15]
In vitro	HT-29 human adenocarcinoma cell line	Pterostilbene (IC_50_, 22.4 μmol/L), resveratrol (IC_50_, 43.8 μmol/L)	P38 mitogen-activated protein kinase cascade	[14]
In vivo	Mouse susceptibility No. 8 (SAMP8) AD model	Pterostilbene was a more effective cognitive and cellular stress regulator than resveratrol	The increasing of the expression of peroxisome proliferator-activated receptor α	[10]
In vivo	Wistar rats fed an obesogenic diet	Pterostilbene is more effective than resveratrol at a dose of 15 mg/kg/d	The decrease of lipogenesis in adipose tissue and the increase of fatty acid oxidation in liver	[88]
In vivo	Wistar rats fed an obesogenic diet	15 mg/kg body weight/d was not as effective as pterostilbene in reducing serum glucose levels	The increase of liver glucokinase activity and skeletal muscle glucose uptake	[93]
In vitro	*Fusobacterium nucleatum*	The minimum inhibitory concentration was more than 60 times lower than that of resveratrol	Inducing the leakage of cell contents, which resulted in the loss of bacterial cell vitality	[95]
